# Creating a palliative care skills training for health care professionals in allogeneic haematopoietic stem cell transplantation recipients: symptom management to end-of-life care

**DOI:** 10.1186/s12904-026-02429-x

**Published:** 2026-11-13

**Authors:** Alinda Reimer, Anne Pralong, Carolin Schepers, Berenike Schoerger, Sukhvir Kaur, Merle Ley, Georg Franke, Michael Hallek, Christoph Scheid, Raymond Voltz, Pusch Lisa, Tim Richardson, Udo Holtick, Steffen T. Simon, Marco Herling

**Affiliations:** 1 Department of Palliative Medicine, Faculty of Medicine and University Hospital Cologne, University of Cologne, Cologne, Germany; 2 Department of Haematology, Cellular Therapy, Haemostaseology, Infectious Diseases and Cancer Centre Central Germany (CCCG) Leipzig-Jena, University of Leipzig, Leipzig, Germany; 3 Faculty of Medicine and University Hospital Cologne, Department I of Internal Medicine, Centre for Integrated Oncology Aachen Bonn Cologne Duesseldorf, Aachen, Bonn, University of Cologne, Cologne, Duesseldorf Germany; 4 Faculty of Medicine and University Hospital Cologne, Centre for Integrated Oncology, University of Cologne, Cologne, Germany; 5 Faculty of Medicine and University Hospital Cologne, Centre for Health Services Research (ZVFK), University of Cologne, Cologne, Germany

**Keywords:** Allogeneic haematopoietic stem cell transplantation, Health care professionals, Palliative care in haematology, Competence training

## Abstract

**Background:**

Allogeneic haematopoietic stem cell transplantation (allo-HSCT) presents a complex procedure with curative intent in haematological disorders, but is associated with high toxicity. Acute graft-versus-host disease (GvHD) and infections are early complications with high mortality, while fatigue and chronic GvHD contribute to significant late morbidity. Our preceding study revealed substantial palliative care needs, including patients’ uncertainty and existential fears, alongside Health Care Professionals (HCP) reported uncertainty and call for further training in addressing these challenges.

**Methods:**

To address these needs, we developed and piloted a one-day training for HCPs, based on Brown’s Design Thinking principles, covering symptom management, end-of-life care, patients’ and family caregivers’ perceived life threat, and goals-of-care discussions. The training, led by a palliative care specialist, was held in three 6-8 h sessions with a total of 28 participants. It was delivered through various learning modules, interactive components, and group discussions. The programme was evaluated at three time points (pre-training, post-training, one-month follow-up) using self-confidence and practice transfer surveys, complemented by open feedback, and analysed descriptively.

**Results:**

At one month post-training, participants reported increased self-confidence across all domains on a 10-point scale (Δmdn = + 1 to + 2), with the most substantial gains in goals-of-care discussions and end-of-life care. Training content was positively evaluated across all HCP groups, and practice transfer was rated as moderate. Feedback highlighted the value of case-based discussions, with calls for more interactive and interdisciplinary elements.

**Conclusions:**

Despite a small sample, single-centre design, and short-term follow-up, this study provides a scalable model for integrating palliative care education into haematology settings. The findings suggest that structured, interdisciplinary training can strengthen HCPs’ confidence and competence in addressing palliative care needs in the allo-HSCT context. Future studies should evaluate the intervention across multiple centres to assess its broader applicability and impact.

**Trial registration:**

It was registered in the German Registry of Clinical Trials (DRKS00027290) on January 10, 2022.

**Supplementary Information:**

The online version contains supplementary material available at https://doi.org/10.1186/s12904-026-02429-x.

## Background

Allogeneic haematopoietic stem cell transplantation (allo-HSCT) signifies a complex, intensive treatment, and is often a last hope for patients with haematological diseases. While potentially curative, it carries significant risks, including those of acute and chronic graft-versus-host disease (GvHD), multiple other chronic organ dysfunctions, infections, sleep disorders, fatigue, as well as symptoms of post-traumatic stress disorders, all contributing to high morbidity and mortality rates [1–6]. Notably, long-term data indicate that 10-year overall survival rate after allo-HSCT remains around 50% across most subgroups [7]. This coexistence of the high curative potential paralleled by the significant risk of mortality throughout the treatment causes high emotional stress in patients, and their family and caregivers.

Despite prolonged symptom burden and complex care needs, concepts for the early integration of specialised palliative care into the allo-HSCT trajectory are still largely lacking [8–11]. Therefore, it becomes crucial that, beyond their focus on clinical patient outcomes, HCPs are better enabled to navigate and manage the patients’ life threat connected to allo-HSCT, where life threat refers to the patients’ awareness of the potential possibility of death in the context of their disease or the transplantation itself, and not to their objective risk of death. Besides an understanding of the medical complications, this requires a high level of knowledge, confidence, and competence in managing palliative scenarios, particularly with respect to symptom management and end-of-life care. Currently, there are very few studies addressing this issue.

Initial findings from our AlloPaS project [12] reveal a uncertainty about disease progression, linked to simultaneous hope for a cure and fear of dying in patients undergoing allo-HSCT. Patients emphasised the importance of open and honest conversations about their life threat, particularly with their haematologist rather than with palliative care specialists [13]. At the same time, the primary haematology care teams expressed a need for greater support and training in the communication of palliative care aspects to navigate these conversations more effectively, such as, by palliative care trained specialists [14, 15]. These findings highlight the importance of targeted interventions to support HCPs in better engaging in palliative care discussions and enhancing their palliative care skills, ultimately benefiting both professionals and patients.

Building on the findings of the AlloPaS project [12], the here presented study sought to develop and evaluate a training programme with a dedicated focus on palliative care to enhance HCPs’ knowledge, competence, and communication skills. Specifically, the programme aimed to increase HCPs’ confidence in addressing the life threat associated with allo-HSCT, symptom management, decision-making, and end-of-life care, thereby promoting optimal supportive palliative care for patients and their family caregivers.

## Methods

The training programme was developed based on the Design Thinking (DT) methodology as proposed by Brown [16]. The DT method introduces a framework for navigating complex challenges, widely embraced across disciplines and is finding increasing application in healthcare [17, 18]. We applied the steps inherent to the DT to evaluate and refine a training programme for HCPs in the context of allo-HSCT care.

Generally, the DT process consists of five steps.

- Step 1 (Empathise): Understanding individuals’ perspectives by observing their needs and burdens.
- Step 2 (Define): Analysing and structuring the identified issues to define a core problem statement.
- Step 3 (Ideate): Developing potential solutions and concepts to address the defined core problem.
- Step 4 (Prototype): Creating a first concept of a selected solution that allows for testing and refinement.
- Step 5 (Test): Gathering feedback and iterating on the prototype to improve the final solution.

**Fig. 1:**
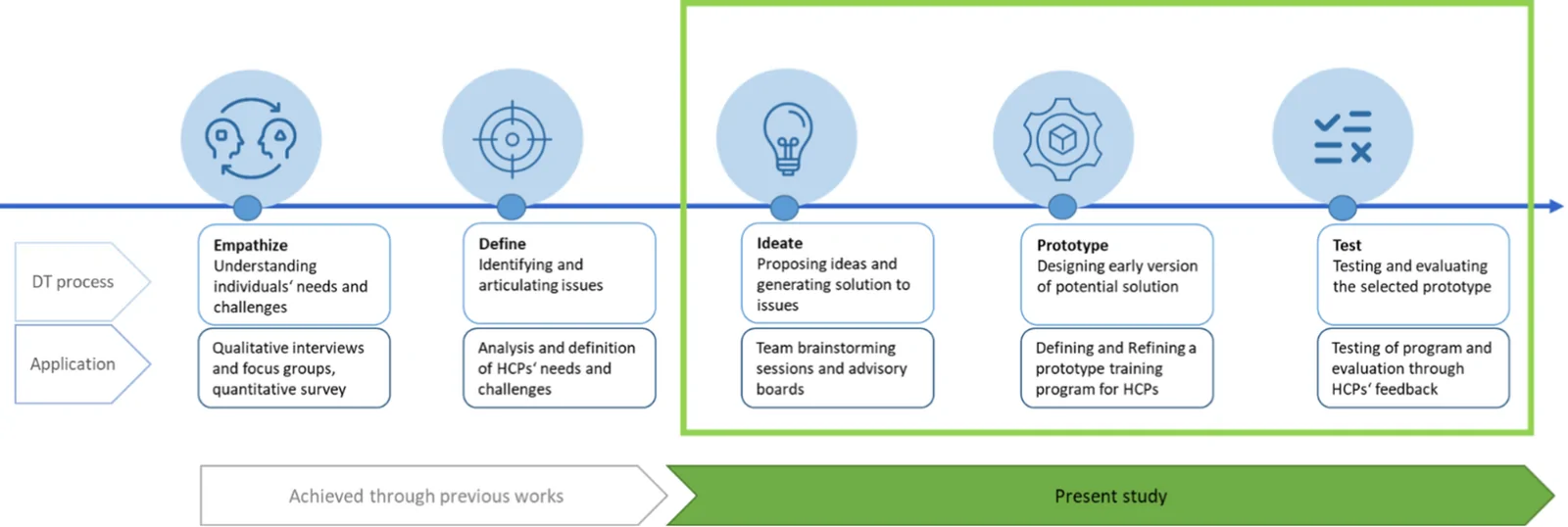
Steps in the development of a training programme based on the DT-methodology

In this paper, we focus specifically on the phases Ideate (Step 3), Prototype (Step 4), and Test (Step 5), while the phases Empathise and Define (Step 1 and 2) have been addressed in previous works [13–15]. Steps 1 and 2 served as a conceptual basis for the subsequent Steps 3–-5 in the development of the intervention. Steps 3-5 represent the practical application of the insights gained in the earlier steps, translating identified challenges and needs into tangible solutions. Figure 1 provides a schematic summary of the applied phases, illustrating the steps of the DT process adapted to the context of this study. Each of these five steps was applied within the project as follows:

The phases Empathise (Step 1) and Define (Step 2) were based on results from previous work we conducted as part of the AlloPaS project [12] on the various, especially palliative-care associated, challenges in the allo-HSCT setting faced by patients, their family caregivers and their HCPs [13–15]. AlloPaS was approved by the Ethics Commission of Cologne University’s Faculty of Medicine (number: 20-1370_2). It included eleven in-depth qualitative interviews and three focus groups with HCPs (including haematologists, nurses, and psycho-oncologists), as well as a cross-sectional survey involving 104 HCPs from the same professional groups. Therein, specific needs for support that arised in managing these complex interactions were identified.

Ideate (Step 3) and Prototype (Step 4): These phases focus on (Step 3) generating innovative solutions to address the specific challenges faced by HCPs and on (Step 4) developing a subset of these ideas through an iterative process. This approach aims to refine and support concepts for interventions that could effectively enhance clinical practice for HCPs.

Through structured brainstorming sessions within the study team and advisory boards jointly defined learning objectives were established and potential educational strategies were evaluated. To address the needs of those involved in allo-HSCT care and to incorporate relevant interdisciplinary expertise, the advisory board included professionals from nursing, haematology, palliative care, psychology, and health services research. Based on the findings of the AlloPaS project, these iterative discussions underwent multiple rounds of refinement, leading to the development of a training programme prototype. This collaborative approach ensured that the resulting educational framework was tailored to the complex demands of allo-HSCT care.

Test (Step 5): The goal of this phase was to refine and validate the outcomes from Step 4 using feedback from HCPs. This involved testing the conceptualised training with HCPs at a single centre and collecting their feedback to enhance and iterate on the prototype based on the insights gained. The pseudonymised feedback collection process was primarily quantitative (via paper-pencil surveys) with a complementary qualitative approach (open-ended questions and a feedback discussion round after the workshop).

**Fig. 2:**
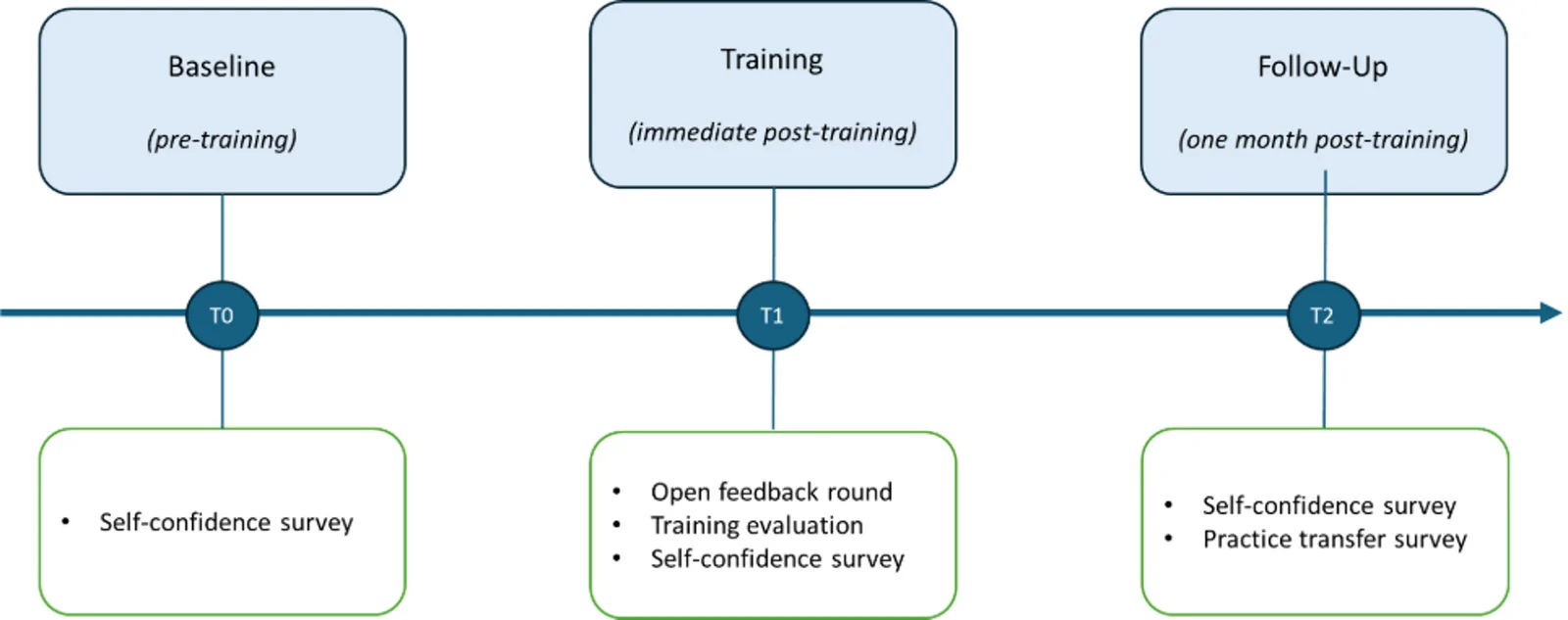
Questionnaires for evaluation of training (T0-T2)

To evaluate the training’s impact and gather meaningful feedback, data were collected at three different time points: T0 (pre-training), T1 (immediately post-training), and T2 (one month post-training). The self-confidence questionnaire was adapted from a previously developed instrument at our centre [19], while the practical relevance and evaluation questionnaires were developed by the study team. As no validated instruments were available for these specific outcomes, the questionnaires were tailored to the objectives of this training programme. At each time point, different self-developed questionnaires were administered (as illustrated in Fig. 2):

- Self-Confidence Survey (T0, T1, T2): This questionnaire assessed participants’ self-perceived confidence in handling the content of the training. Items were rated on a 10-point Likert scale (1 = not at all, 10 = very much) (Supplementary Material 1)
- Training Evaluation Survey (T1): This questionnaire contained general questions regarding the evaluation and potential improvements for the training programme and its topics. Responses to all questions were rated on a 5-point Likert scale (1 = not at all, 5 = very much) (Supplementary Material 2)
- Practice Transfer Survey (T2): This questionnaire assessed the practical applicability and self-perceived confidence in applying the workshop’s content. It included both open- and closed-ended questions, with closed questions rated on a 5-point Likert scale (1 = not at all, 5 = very much) (Supplementary Material 3)

At T1 (immediately post-training), additional qualitative feedback was gathered through a structured feedback discussion round. The qualitative responses were documented and analysed by identifying recurring topics.

This approach enabled structured feedback collection in a controlled environment to gather structured feedback on the training, providing valuable insights into its strengths and areas for improvement. The feedback collected through the evaluation played a critical role in refining the training content and its delivery to better meet the needs of the target group.

Quantitative data from the three self-developed questionnaires were analysed descriptively using Median (Mdn) and Interquartile Range (IQR) to assess changes over time (T0, T1, T2). No composite or subscale scores were calculated. Given the exploratory nature of the study and the limited sample size, no inferential statistics were applied. Qualitative data obtained through open-ended questions and a moderated group feedback session were summarised by identifying recurring topics. Recurring themes were identified inductively from the protocol notes and grouped into thematic categories through collaborative team discussion among an interdisciplinary team consisting of two psychologists (C.S., M.L.) and one health services researcher (A.R.), all experienced in qualitative research. Summaries remained close to participants’ original wording to preserve meaning. No audio recording was made, as the small group size and interpersonal familiarity among participants would have risked inhibiting honest feedback. Responses were documented in an anonymised form. The qualitative findings served to contextualise and deepen the interpretation of the quantitative results, particularly regarding the practical application and perceived effectiveness of the training programme.

## Results

The outcomes of this study emerged through systematic application of the DT process, as outlined in the Methods section. Key outcomes from the Ideate (Step 3), Prototype (Step 4), and Test (Step 5) phases of the DT process are presented here.

An interdisciplinary advisory board supported the development process, bringing together diverse expertise essential for allo-HSCT care. The board was composed of two allo-HSCT nurses, three haematologists, one palliative care specialist, two psychologists, and two health services researchers. This composition ensured that both clinical and psychosocial perspectives were integrated into the design of the educational intervention, reflecting the complex needs of patients and the multidisciplinary nature of their care.

Ideate (Step 3): This phase focused on generating solutions tailored to address the communication and emotional challenges of HCPs working in allo-HSCT. Through the described iterative brainstorming sessions in an interdisciplinary team, several innovative training components were formulated and defined as key interventions:

- Simulation-based training using real-life scenarios to strengthen (palliative) communication skills, based on authentic clinical communication situations reflecting a range of routine and complex care encounters. These scenarios were drawn from real clinical situations, such as those regularly encountered on allogeneic stem cell transplant units. The interdisciplinary project team selected them to cover both routine and complex communication challenges. One example was the following: a patient with a complicated post-transplant course and severe symptoms, whose family member repeatedly inquired about further treatment options. Working in small groups, the participants assumed clinical roles, such as nurse, resident, or attending physician. Based on the assigned scenario, they role-played a brief bedside conversation around goals of care. This was followed by a brief role swap and a structured group debriefing to reflect on the communication strategies used.
- Interactive group discussions centred on practical case studies to foster peer learning and real-world application. Without engaging in direct role-playing, the participants were encouraged to discuss specific aspects here. For example, the participants reflected together on which emotions, expressed by patients or family members, they found difficult to handle, what made these situations particularly challenging, and what helped them to deal with these feelings in clinical practice.
- Modular, evidence-based learning units designed to address aspects of palliative care and communication with patients and their family caregivers.

Prototype (Step 4): Based on the preceding phases, the study team and advisor board decided to implement and pilot the training in a one-day, single-centre format, led by a palliative care specialist. These sessions were conducted as one-day training workshops lasting 6-8 h each. The prototypic training programme focused on key topics: symptom management, end-of-life care, addressing the life threat with the patients and their family caregivers and goals of care discussions. The training comprised a variety of educational formats, such as lectures, Q&A sessions, practical exercises, role-play activities, and group discussions. The structure of the prototyped training programme is outlined in Table 1.

**Table 1:** Description of the training programme

Module	Content	Learning	Teaching Methods
1. Introduction (plan, content, expectations)	Expectations, project description, content and goals of workshop	Knowledge	Group discussion
2. Symptom management	Pain, nausea/vomiting, dyspnea, fatigue, insomnia, anxiety	Knowledge	Lecture
3. End-of-life	Recognising the dying phase, what good end-of-life care looks like, medications, measures after death, team reflection on challenges and best practices	Knowledge	Lecture, Q&A session, exercise
4. Handling life threat / fear of dying	What is it about?, recognising & perceiving, attitude & behaviour, communication (tips & tools, seeking support)	Skills	Lecture, two exercises
5. Determining goals of care	Fundamentals & key concepts, challenges in goals-of-care discussions, proposed SCT pathway for treatment goal clarification, exercise, add-on: breaking bad news	Skills	Lecture, role play exercise
6. Family Caregivers	Communication & tools, exercise: barriers and facilitators in working with family caregivers	Knowledge	Lecture, partnered reflection
7. Feedback (including qualitative & quantitative survey)	--	Reflection	Assessment
8. Conclusion & closing	--	Reflection	Group discussion

Test (Step 5): A total of 28 participants (5 physicians and 23 nurses, including 4 HSCT coordinators) took part in three separate workshops at the University Hospital of Cologne. 71.43% of participants were female. Training was facilitated by a senior physician (author S.T.S.) with palliative care expertise.

Based on the training evaluation survey (T1), the training was generally well received across all HCP groups, with high ratings for overall structure, content clarity, and relevance. Participants rated the opportunity to ask questions as very positive on the 5-point Likert scale (Mdn = 5, IQR = 1), as well as the opportunity to share their own experiences and discuss relevant topics (Mdn = 4, IQR = 2). At the same time, the responses to the question of whether they could freely express their own opinions or share their own experiences (M = 5, IQR = 1) experiences, and whether they were satisfied with the organization and execution, were positive (M = 5, IQR = 1).

The training materials were also rated as supportive (Mdn = 5, IQR = 1). Content was perceived as clearly conveyed across all domains, including symptom management, end-of-life care, and addressing life threat/death-related anxiety (all Mdn = 5, IQR = 1).

Descriptive analysis of the self-confidence survey (T0-T2), which assessed participants’ self-perceived confidence in managing training-related content on a 10-point Likert scale (1 = not at all, 10 = very much), revealed a continuous increase in participants’ self-reported confidence across all assessed domains from T0 (pre-training) to T2 (one-month post-training). The overall development was positive, with the most substantial improvements observed in changes in goals of care and end-of-life care (both Δmdn = + 2). See Table 2.

**Table 2:** Changes in self-confidence across domains (T0–T2)

Category	T0 (*n* = 26)	T1 (*n* = 24)	T2 (*n* = 14)	T0–T2 Change
General perception of handling challenging conversations	7 [2]	7,5 [1]	8 [1]	+ 1
Changes in goals of care	6 [2]	7 [2]	8 [1]	+ 2
Addressing life threat / death-related anxiety	6 [3]	8 [3]	7,5 [2]	+ 1,5
Symptom management	7 [3]	7 [2]	8 [2]	+ 1
End-of-life care	6 [3]	7 [3]	8 [2]	+ 2

Values presented as mean ± standard deviation

Concerning the practice transfer survey (T2), HCPs rated the successful application of acquired knowledge in clinical practice at Mdn = 3 (IQR = 1), suggesting a moderate transfer of training content into clinical routines. Regarding the integration of specific tools, methods, and materials (such as, SPIKES [20], NURSE [21]) introduced during the training, participants rated their implementation with at Mdn = 3 (IQR = 1).

Open-ended questions indicated that the training was experienced to enhance awareness and competence. However, HCPs emphasised the need for more interdisciplinary exchange, case-based discussions, and hands-on practice to reinforce long-term impact. To further improve the training, HCPs suggested shifting away from lecture-based formats in favour of interactive and interdisciplinary approaches, increasing case study integration, and providing practical discussions on goals-of-care implementation.

**Fig. 3:**
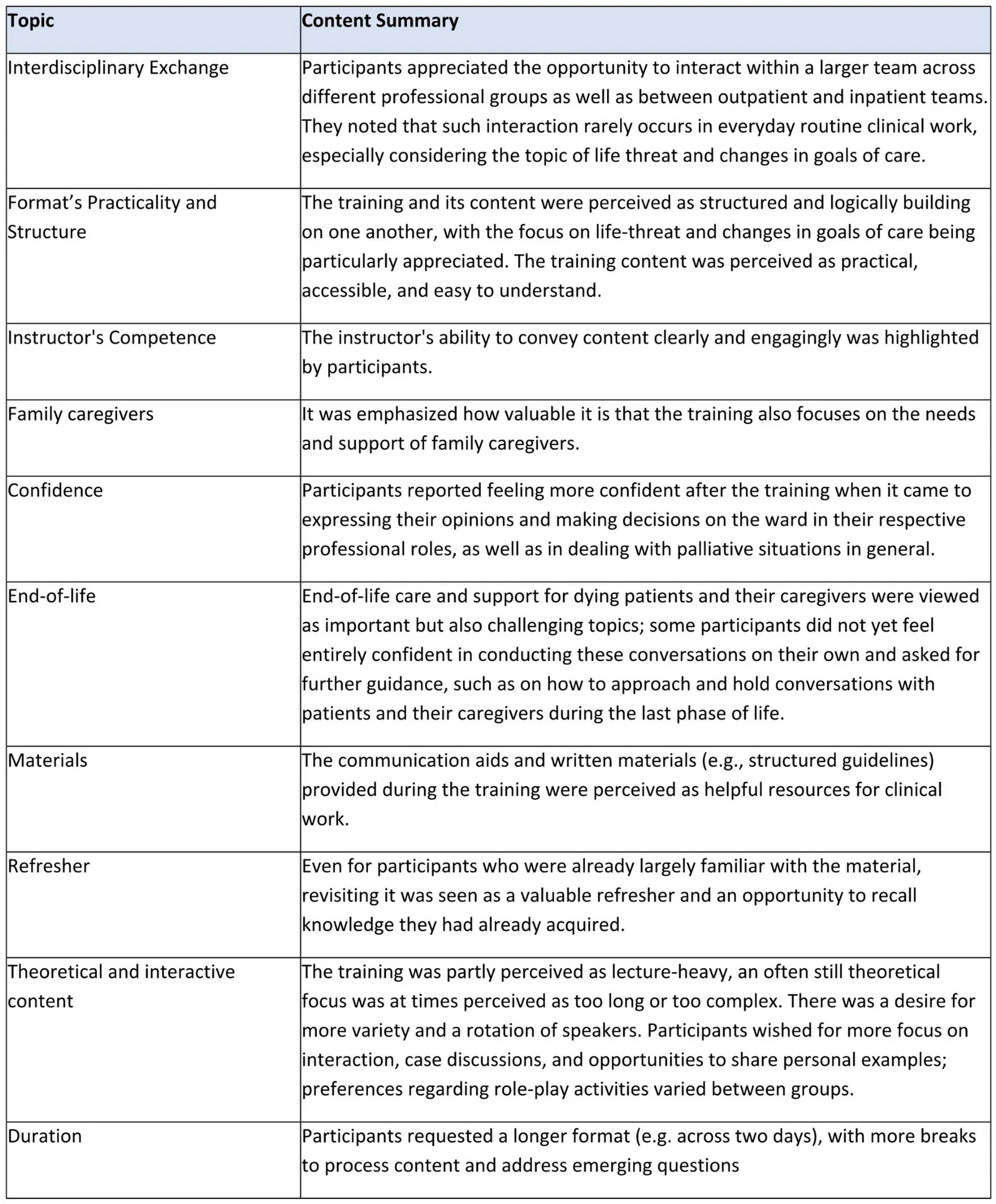
Summary of themes in feedback discussions

Qualitative feedback from HCPs immediately after the training (T1) indicated that they perceived the training to be well-structured and highly relevant to their clinical practice, particularly appreciating its focus on changes in goals of care, patients’ life threat, and end-of-life care. They emphasised the practical value of real-life case discussions but expressed a need for more interactive elements, such as role-playing, and extended time for complex topics (Fig. 3).

## Discussion and conclusion

Our training is designed to enhance HCPs’ skills in palliative and supportive care as well as communication, specifically tailored to the unique challenges of allo-HSCT patients. The focus on symptom management, end-of-life issues, patients’ and family caregivers’ perception of life threat, as well as goal-of-care discussions reflects the needs identified in the AlloPaS-project’s earlier phases. Findings from our single-centre pilot confirm and emphasise the training topics and suggest that the training strengthens participants’ competence and confidence in addressing these sensitive topics.

The combination of lectures, Q&A sessions, role-play, and case-based group discussions has proven beneficial in preparing HCPs to navigate emotionally complex situations and communicate more openly with patients and their family caregivers. Beyond knowledge acquisition, the training experience emphasises the importance of encouraging HCPs to take initiative in initiating conversations around palliative needs, to actively involve palliative care specialists when needed, whether through coaching or case-specific consultations, and to more systematically integrate palliative care expertise into team structures.

Palliative care can support the understanding of prognosis and treatment, fostering effective coping and facilitating advanced care planning in oncology patients [22]. Early palliative care interventions have been found to improve symptom management and quality of life [23–25] and are recommended as standard of care for patients with advanced solid tumours in international guidelines [26–29]. Extending these findings to haematological malignancies, El-Jawahri et al. [30] highlighted that inpatient palliative care during allo-HSCT reduced depression and anxiety while improving patients’ quality of life. However, our earlier study [13] indicates that while patients with allo-HSCT felt existentially threatened by the unpredictable course of their illness, patients rarely sought support from specialised palliative care services (such as, hospital palliative teams). Instead, patients view their primary haematologists and nurses as the primary point of contact for addressing fears related to the life threat, with palliative care specialists seen as secondary, with supportive roles when needed. This aligns with the integrative review by Short et al. [9], who emphasise the unmet palliative needs of allo-HSCT patients and the persistent misconceptions about palliative care, highlighting the importance of earlier integration into the transplantation process to enhance quality of life and mitigate the severe consequences of the transplantation. Confirming this, Nelson et al. [31] present that structured palliative interventions during transplantation could improve patients’ mood and quality of life, beyond symptom control alone. Similarly, Lin et al. [32] underscore the importance of timely end-of-life care after transplantion relapse, supporting the need for integrated palliative care.

The AlloPaS study reveals that HCPs are tasked with balancing curative goals while mitigating therapy risks and preserving patients’ hope [14, 15]. The unpredictable nature of individual disease trajectories and the accompanied suffering of patients have placed a significant burden on HCPs, as reflected in both the qualitative and quantitative results of the AlloPaS surveys [13–15]. Also, Hui et al. [33] show that haematologists, compared to solid tumour specialists, more often perceive disease progression as a personal failure and feel more insecure in end-of-life discussions. This highlights the challenge of balancing cure with the uncertain course of haematological malignancies as well as the importance of palliative care training for haematology-oncology teams​.

Other studies confirm that oncologists view conversations around palliative care or the potential end of life as distressing, accompanied by a fear of the impact on patients, such as, of destroying patients’ hope [34] and to cause additional stress to them [35] or feeling inadequately trained or unaccustomed [36–39]. Bennardi et al. [40] underscore that HCPs face barriers to integrating palliative care, including limited awareness, communication challenges, emotional strain, and deeply seated cultural misconceptions, all of which contribute to delays in early utilisation. Breaking bad news is a distressing and isolating experience for practitioners, often exacerbated by a culture of invulnerability, highlighting the need for organisational support, reflective practice and more time for debriefing after difficult conversations [14, 41].

In addition to knowledge transfer, the interprofessional exchange within the team appeared particularly beneficial within the training programme, as it enhanced the ability to articulate perspectives and better understand those of others, which has also been highlighted in previous research [42–44]. Therefore, such exchanges should not only be limited to workshops, but could be systematically integrated into clinical practice such as, by weekly multiprofessional team meetings which include palliative care specialists.

A Cochrane review by Moore et al. confirms that structured communication training enhances supportive interactions with patients [45]. Interventions to enhance communication about end-of-life issues in clinical care must be tailored to the specific challenges HCPs face, especially concerning the complex demands inherent to the described allo-HSCT setting.

We conducted this study to develop and evaluate a training programme specifically aimed at enhancing palliative care skills in allo-HSCT professionals. However, it is important to acknowledge several limitations. Our findings are based on a small, single-centre sample, and the evaluation included only a limited number of HCPs working within a specific institutional context. The landscape of palliative care (regarding available resources, institutional culture, and patient demographics) can vary considerably across centres, even within one country, making it difficult to recommend a universally applicable intervention. Moreover, the study lacked a control group, which limits the ability to assess the training’s effectiveness in comparison to standard practice. As a pilot study, the small sample size limits the generalisability of our results. Additionally, possible ceiling effects observed in the training evaluation might limit the interpretation of satisfaction levels. The proportion of female participants (71.4% across all HCP subgroups) is likely due to the sample composition, which mainly consisted of nurses (23/28 participants), a predominantly female profession in Germany, and fewer physicians (5/28 participants). This may limit generalisability across both professional groups and gender. All outcomes are self-reported by HCPs, which may introduce bias. It would be valuable to complement these findings with patient-reported experiences, particularly concerning satisfaction with communication. A patient follow-up could offer important insights into the perceived impact of the training. In future studies, it will also be crucial to evaluate the sustainability of training effects longitudinally, such as, through an additional measurement point one year post-intervention, to assess whether changes in attitudes and practices persist beyond the immediate post-training period.

Against the background of these limitations, our pilot study has generated promising initial directions to further expand the training approach. Despite local differences in care structures, there is clear potential to adapt and evaluate the intervention in other settings, thereby contributing to the broader integration of palliative-supportive care in haematological oncology in a national rollout.

## Supplementary Information

Supplementary Material 1.

Supplementary Material 2.

Supplementary Material 3.

## Data Availability

Due to the small sample size and the risk of re-identification, the data are not publicly available. De-identified data may be shared upon reasonable request.

## Abbreviations

allo-HSCT: Allogeneic Haematopoietic Stem Cell Transplantation
GvHD: Graft-versus-Host Disease
HCPs: Health Care Professionals
DT: Design Thinking
M: Mean values
SD: Standard Deviations
DJCLS: Deutsche José Carreras Leukämie-Stiftung
