# Supplementary material for: Creating a palliative care skills training for health care professionals in allogeneic haematopoietic stem cell transplantation recipients: symptom management to end-of-life care
Source: BMC Palliat Care. 2026 Nov 13;25:345. doi: 10.1186/s12904-026-02429-x (PMC13760803; doi:10.1186/s12904-026-02429-x)
Supplement: Supplementary file 1 [file 12904_2026_2429_MOESM1_ESM.pdf]

This is a working translation of the original German text, prepared by the authors with the assistance of DeepL and reviewed for accuracy; it has not been formally validated.

Items: Questionnaire Self Confidence (AlloPaS Training Program)

**How confident do you feel…**	Not at all									A lot
	1	2	3	4	5	6	7	8	9	10
**In General**										
…in general when dealing with challenging conversation situations in a professional context?	○	○	○	○	○	○	○	○	○	○

**How confident do you feel…**	Not at all									A lot
	1	2	3	4	5	6	7	8	9	10
**Changes in Goals of Care**										
…in distinguishing between treatment goals, medical indications, and the patient's wishes?	○	○	○	○	○	○	○	○	○	○
…in implementing the process of changing treatment goals?	○	○	○	○	○	○	○	○	○	○
…in a conversation with a patient about changing the treatment goal?	○	○	○	○	○	○	○	○	○	○
…in a conversation with a family member about changing the treatment goal?	○	○	○	○	○	○	○	○	○	○
…in conversations with patients who have different treatment expectations or goals?	○	○	○	○	○	○	○	○	○	○
…in communicating a clear plan for implementing treatment goals to the patient?	○	○	○	○	○	○	○	○	○	○
…when delivering bad news?	○	○	○	○	○	○	○	○	○	○
……how to deal with emotions during discussions about changing goals of care?	○	○	○	○	○	○	○	○	○	○

**How confident do you feel…**	Not at all									A lot
	1	2	3	4	5	6	7	8	9	10
**Changes in Goals of Care**										
… how to deal with the emotions of the person you're talking to (patient/client) regarding a change in therapy goals?	○	○	○	○	○	○	○	○	○	○
… how to deal with the emotions of colleagues regarding a change in therapy goals?	○	○	○	○	○	○	○	○	○	○
… what if someone else confronts you with a change in therapy goals?	○	○	○	○	○	○	○	○	○	○
… how to handle advance directives and powers of attorney (in terms of what you need to keep in mind)?	○	○	○	○	○	○	○	○	○	○

**How confident do you feel…**	Not at all									A lot
	1	2	3	4	5	6	7	8	9	10
**Dealing with life-threat/fear of dying**										
…talking to a patient about the life-threatening nature of their illness?	○	○	○	○	○	○	○	○	○	○
…talking to a patient about their fear of dying?	○	○	○	○	○	○	○	○	○	○
…talking to a family member about the life-threat of the patient's illness?	○	○	○	○	○	○	○	○	○	○
…talking to a family member about their fear of the patient's death?	○	○	○	○	○	○	○	○	○	○
…recognizing and addressing a patient's emotions (e.g., fear, sadness, anger, etc.) related to the life-threat of their illness?	○	○	○	○	○	○	○	○	○	○
…having open and honest conversations about the life-threat of the illness and the dying process?	○	○	○	○	○	○	○	○	○	○

**How confident do you feel…**	Not at all 1	2	3	4	5	6	7	8	9	A lot 10
**Symptom Relief**										
…in managing symptoms in general?	○	○	○	○	○	○	○	○	○	○
…in managing pain?	○	○	○	○	○	○	○	○	○	○
…in managing shortness of breath?	○	○	○	○	○	○	○	○	○	○
…in managing nausea/vomiting?	○	○	○	○	○	○	○	○	○	○
…in managing or supporting patients with anxiety (as a symptom)?	○	○	○	○	○	○	○	○	○	○
…in the use of medications (e.g., which ones? Dosage?) for symptom relief?	○	○	○	○	○	○	○	○	○	○
…specifically in the use of opioids (e.g., morphine, hydromorphone, fentanyl, etc.)?	○	○	○	○	○	○	○	○	○	○
…in symptom relief for patients in the terminal phase?	○	○	○	○	○	○	○	○	○	○

**How confident do you feel…**	Not at all 1	2	3	4	5	6	7	8	9	A lot 10
**End-of-life care**										
…in the treatment and support of patients in the terminal phase?	○	○	○	○	○	○	○	○	○	○
…in the treatment and support of families in the terminal phase?	○	○	○	○	○	○	○	○	○	○
…recognizing and addressing the patient's emotions (e.g., fear, sadness, anger, etc.) related to the end-of-life phase?	○	○	○	○	○	○	○	○	○	○

This is a working translation of the original German text, prepared by the authors with the assistance of DeepL and reviewed for accuracy; it has not been formally validated.

**How confident do you feel…**	Not at all									A lot
	1	2	3	4	5	6	7	8	9	10
**End-of-life care**										
…when deciding on medications and treatments (which should be discontinued or stopped, and which are still indicated or appropriate) at the end of life?	○	○	○	○	○	○	○	○	○	○
…when discussing a likely imminent death with the patient themselves?	○	○	○	○	○	○	○	○	○	○
…when discussing a likely imminent death of patients with their relatives?	○	○	○	○	○	○	○	○	○	○
…in dealing with one's own fear of dying (regarding one's own mortality)?	○	○	○	○	○	○	○	○	○	○
…in dealing with grief?	○	○	○	○	○	○	○	○	○	○
