# Supplementary material for: Creating a palliative care skills training for health care professionals in allogeneic haematopoietic stem cell transplantation recipients: symptom management to end-of-life care
Source: BMC Palliat Care. 2026 Nov 13;25:345. doi: 10.1186/s12904-026-02429-x (PMC13760803; doi:10.1186/s12904-026-02429-x)
Supplement: Supplementary file 2 [file 12904_2026_2429_MOESM2_ESM.pdf]

This is a working translation of the original German text, prepared by the authors with the assistance of DeepL and reviewed for accuracy; it has not been formally validated.

Items: Questionnaire for the Evaluation (AlloPaS Training Program)

	1 (not at all)	2	3	4	5 (very much)
**General**					
Were there enough opportunities to ask questions during the training?	○	○	○	○	○
Was the training long enough?	○	○	○	○	○
Were there enough opportunities to discuss topics that were relevant to you?	○	○	○	○	○
Were you able to freely express your own opinions and experiences?	○	○	○	○	○
Did the materials (slides, etc.) effectively support the training content?	○	○	○	○	○
Were you satisfied with the organization and delivery of the training (facilities, scheduling, catering)?	○	○	○	○	○

	1 (not at all)	2	3	4	5 (very much)
**Clarifying or Changing Goals of Care**					
Did the training help you improve your ability and confidence in changing of goals of care?	○	○	○	○	○
Were you able to learn strategies during the training that will help you better handle challenges related to changing of goals of care in the future?	○	○	○	○	○
How clear was the content on the topic of changing of goals of care?	○	○	○	○	○
How clear were the materials and slides on the topic of changing of goals of care?	○	○	○	○	○

Other: Did you take away anything valuable or helpful regarding the clarification or modification of goals of care? Was there anything you felt was missing? Which subtopics should be covered in more detail, and which ones can be addressed more briefly?

This is a working translation of the original German text, prepared by the authors with the assistance of DeepL and reviewed for accuracy; it has not been formally validated.

	1 (not at all)	2	3	4	5 (very much)
**Dealing with life-threat/Fear of dying**					
Did the training help you improve your skills and confidence when dealing with the topic of "life-threat situations and the fear of dying"?	○	○	○	○	○
Were you able to learn strategies during the training that will help you better handle challenges related to "life-threat and the fear of dying" in the future?	○	○	○	○	○
How clear was the content on the topic of "life-threat and the fear of dying"?	○	○	○	○	○
How clear were the materials and slides on the topic of "life-threatening situations and the fear of dying"?	○	○	○	○	○

Other: Did you take away anything valuable or helpful regarding the life threat and the fear of dying? Was there anything you felt was missing? Which subtopics should be covered in more detail, and which ones can be addressed more briefly?

	1 (not at all)	2	3	4	5 (very much)
**Symptom relief**					
Did the training help you improve your skills and confidence in managing symptoms?	○	○	○	○	○
Were you able to learn strategies during the training that will help you better handle challenges related to symptom management in the future?	○	○	○	○	○
How clear was the content on symptom management?	○	○	○	○	○
How clear were the materials and slides on symptom management?	○	○	○	○	○

This is a working translation of the original German text, prepared by the authors with the assistance of DeepL and reviewed for accuracy; it has not been formally validated.

Did you take away anything valuable or helpful for managing symptoms? Was there anything you felt was missing? Which subtopics should be covered in more detail, and which ones can be addressed more briefly?

	1 (not at all)	2	3	4	5 (very much)
**End-of-life care**					
To what extent did the training help you improve your skills and confidence in end-of-life care?	○	○	○	○	○
Were you able to learn strategies during the training that will help you better handle challenges related to end-of-life care in the future?	○	○	○	○	○
How clear was the content on end-of-life care?	○	○	○	○	○
How clear were the materials and slides on end-of-life care?	○	○	○	○	○

Did you take away anything valuable or helpful regarding end-of-life care? Was there anything you felt was missing? Which subtopics should be covered in more detail, and which ones can be addressed more briefly?

**All Topics**					
Which topic can be covered more briefly?	○ Changes in goals of care	○ Life Threat/Fear of Dying	○ Symptom Relief	○ End-of-life care	○ None
Which topic should be covered at greater length or in more detail?	○ Changes in goals of care	○ Life Threat/Fear of Dying	○ Symptom Relief	○ End-of-life care	○ None

Was there anything you liked about the training?

This is a working translation of the original German text, prepared by the authors with the assistance of DeepL and reviewed for accuracy; it has not been formally validated.

○ Yes ○ No

If yes, what?

Is there anything else you can share with us to help us improve the training? Any other feedback?
