# Supplementary material for: Creating a palliative care skills training for health care professionals in allogeneic haematopoietic stem cell transplantation recipients: symptom management to end-of-life care
Source: BMC Palliat Care. 2026 Nov 13;25:345. doi: 10.1186/s12904-026-02429-x (PMC13760803; doi:10.1186/s12904-026-02429-x)
Supplement: Supplementary file 3 [file 12904_2026_2429_MOESM3_ESM.pdf]

This is a working translation of the original German text, prepared by the authors with the assistance of DeepL and reviewed for accuracy; it has not been formally validated.

Items: Questionnaire on Practice Transfer (AlloPaS Training)

	1 (not at all)	2	3	4	5 (very much)
**Practical Application**					
How confident did you feel in applying the topics covered during your daily work?	○	○	○	○	○
To what extent have you successfully applied the knowledge gained in the training to your daily work?	○	○	○	○	○
How well were you able to integrate the tools, resources, and handouts from the training into your daily work?	○	○	○	○	○

**Changes in Goals of Care**		
Have you encountered the issue of changing of goals of care in your daily clinical practice?	○<br>○	Yes<br>No
If yes: Were you able to apply what you learned in the training?	○<br>○	Yes<br>No
If yes: Was the training content helpful?	○<br>○	Yes<br>No

Why was this helpful or unhelpful? Please explain in detail.

**Dealing with life-threat/fear of dying**		
Did the topic of dealing with life-threat or the fear of dying come up in your daily clinical practice?	○<br>○	Yes<br>No
If yes: Were you able to apply what you learned in the training?	○<br>○	Yes<br>No
If yes: Was it helpful?	○<br>○	Yes<br>No

What specific improvements have you noticed in your interactions with patients and their families in your clinical practice since completing the training?

This is a working translation of the original German text, prepared by the authors with the assistance of DeepL and reviewed for accuracy; it has not been formally validated.

**Symptom Relief**		
Did the topic of symptom relief come up in your daily clinical practice?	○<br>○	Yes<br>No
If yes: Were you able to apply what you learned in the training?	○<br>○	Yes<br>No
If yes: Was it helpful?	○<br>○	Yes<br>No

What specific improvements have you noticed in your interactions with patients and their families in your clinical practice since completing the training?

**End-of-life-Care**		
Did the topic of end-of-life care come up in your daily clinical practice?	○<br>○	Yes<br>No
If yes: Were you able to apply what you learned in the training?	○<br>○	Yes<br>No
If yes: Was that helpful?	○<br>○	Yes<br>No

What specific improvements have you noticed in your interactions with patients and their families in your clinical practice since completing the training?

Was there a tool that you used particularly often in your day-to-day work?

If you haven't used a particular tool, which one and why not?

This is a working translation of the original German text, prepared by the authors with the assistance of DeepL and reviewed for accuracy; it has not been formally validated.

Did the training have any other impact on your daily clinical practice? If so, what kind?

Now that you've had a chance to apply what you learned in the training in a real-world setting, if you were to look back on the training: Do you have any suggestions for improving it?

Suppose you received a donation of €1,000,000 to bring about a significant improvement in how your department addresses the issue of "life-threat/fear of dying." Where would you invest this money? (Possible ideas: investment in resources, e.g., training, etc.; investment in personnel: for which profession(s) and with which role(s)?)
